# Oxidizing pollutants can disrupt nestmate recognition in ants

**DOI:** 10.1073/pnas.2520139123

**Published:** 2026-02-02

**Authors:** Nan-Ji Jiang, Bhoomika Ashok Bhat, Eduardo Briceño-Aguilar, Angela Lehmann, Yuko Ulrich, Bill S. Hansson, Markus Knaden

**Affiliations:** ^a^Department of Evolutionary Neuroethology, Max-Planck Institute for Chemical Ecology, Jena 07745, Germany; ^b^Max Planck Center Next Generation Insect Chemical Ecology, Jena 07745, Germany; ^c^Shenzhen Branch, Guangdong Laboratory of Lingnan Modern Agriculture, Key Laboratory of Synthetic Biology, Ministry of Agriculture and Rural Affairs, Agricultural Genomics Institute at Shenzhen, Chinese Academy of Agricultural Sciences, Shenzhen 518000, China; ^d^Social Behaviour Group, Max-Planck Institute for Chemical Ecology, Jena 07745, Germany; ^e^Workshop, Max-Planck Institute for Chemical Ecology, Jena 07745, Germany

**Keywords:** cuticular hydrocarbons, eusociality, pollution, Anthropocene

## Abstract

Eusocial hymenoptera recognize nestmates based on colony-specific profiles of cuticular hydrocarbons (CHCs). While these profiles contain a variety of alkanes, the less abundant alkenes are crucial for nestmate recognition [F. R. Dani *et al.*, *Chem. Senses* **30**, 477–489 (2005)]. However, due to their carbon–carbon double bonds, alkenes can easily become degraded by oxidants. One of those oxidants, ozone, that usually reaches concentrations of 10 ppb in nonurban areas and ca 30 ppb in cities nowadays has been reported to often exceed 100 ppb in highly polluted regions, with levels above 200 ppb occasionally reported. Here, we show that short exposure to such elevated levels degrades alkenes in CHC profiles of all six investigated ant species and compromises nestmate recognition in five of them. Furthermore, a separate experiment with long-term exposure to such ozone levels corrupted brood care behavior within ant colonies, resulting in the death of larvae. The oxidizing pollutants like ozone have already been shown to corrupt interactions of flowers and their pollinators [B. Cook *et al.*, *J. Chem. Biol.* **46**, 987–996 (2020)] as well as sex pheromone communication in multiple species of flies [N. J. Jiang *et al.*, *Nat. Commun.* **14** (2023)]. Our data suggest that the detrimental effects of oxidant pollutants may be even more far-reaching by jeopardizing the functionality of eusocial colonies.

## Significance

Ants make up to two-thirds of the biomass of all insects and have efficiently colonized most parts of the world. One reason for their success is likely their social structure. Ants can distinguish nestmates from non-nestmates based on colony-specific cuticular hydrocarbons on their bodies. In the Anthropocene, the amount of oxidant pollutants, such as ozone, in the atmosphere has increased. Here, we demonstrate that even slight increases in ozone levels can degrade some of the ants’ hydrocarbons, thereby negatively impacting nestmate recognition in numerous ant species.

Social behavior represents a successful evolutionary strategy for animals to adapt to dynamic environments. While sociality is broadly observed across life forms, eusociality—characterized by advanced division of labor, cooperative care of offspring, and overlapping generations—has evolved in certain organisms including ants, bees, wasps, aphids, and even mammals like naked mole rats (1, 2). Eusocial insects are known to utilize diverse mechanisms to share information and sustain their social structures, with chemical communication playing a fundamental role (3).

In ants, cuticular hydrocarbons (CHCs) play an important role in governing nestmate recognition and division of labor (4, 5). These CHCs, which are usually long-chain molecules, likely originated as adaptations to prevent desiccation but since then have evolved to function as social recognition cues in many species (6–9). The qualitative composition of CHC profiles often is species-specific, while between conspecific colonies the quantity of individual compounds of the profile can vary dramatically (10). Colony-specific profiles allow ants to effectively distinguish their nestmates from non-nestmates. The ants achieve diversity in their CHC profiles through biochemical modifications, such as methyl branching or the addition of carbon–carbon double bonds, resulting in alkenes (4). Because the double bond is a target for oxidative agents, we ask whether nestmate recognition and division of labor in ants might be compromised by increasing levels of oxidizing pollutants due to the Anthropocene.

Human activities, including industrial and agricultural emissions, combined with global climate change, have led to elevated levels of atmospheric oxidants like ozone and nitrogen oxides (11). Ozone concentrations are around 10 ppb in nonurban areas and commonly reach 30 ppb in cities, often exceeding 100 ppb in highly polluted regions, with levels above 200 ppb occasionally reported (12–16). While the impact of ozone on human health is well documented (17), its effects on animals is less understood, yet of great importance. Carbon–carbon bonds degrade even at background ozone levels and more rapidly under elevated ozone, potentially compromising all communication channels based on unsaturated compounds. Many olfactory cues that drive attraction of insect pollinators to flowers contain unsaturated compounds. As a result, increased levels of ozone have been shown to hinder insect foraging by altering plant volatiles (18–21). At the same time, the majority of insect pheromones identified to date contain carbon–carbon double bonds (22). Consequently, even slightly elevated levels of ozone can disrupt partner recognition by oxidizing pheromones (23, 24), and thereby potentially could contribute to the decline of insect populations (25).

In this study, we show that elevated levels of ozone, probably by oxidizing the alkenes of ants’ CHC profiles, can in addition impair nestmate recognition in ants and their division of labor. Given that we found compromising effects of ozone in all six investigated ant species belonging to the subfamilies Formicinae, Myrmicinae, and Dorylinae our study suggests that oxidizing pollutants such as ozone potentially could have significant effects on the social structure of many eusocial insects.

## Ozone Exposure Elicits Nestmate Aggressive Behavior in *M. barbarus*.

**Fig. 1.**
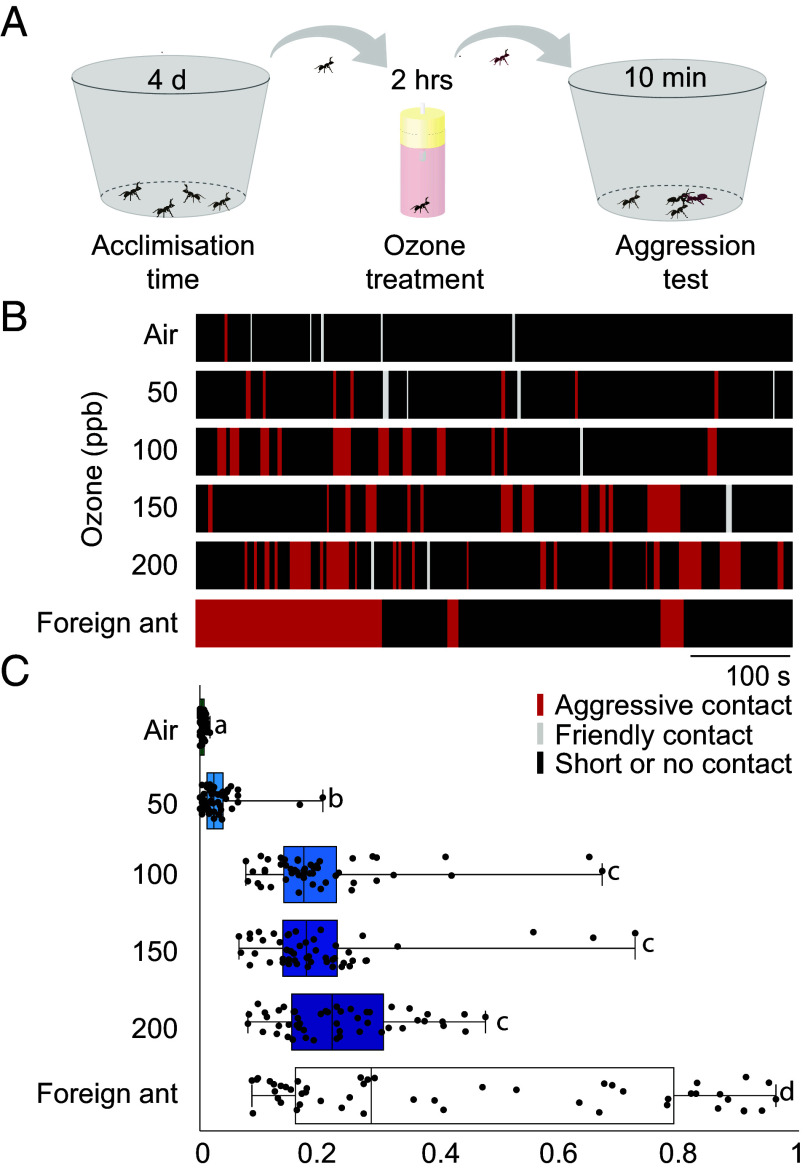
Ozone exposure elicits aggressive behaviors between nestmates in *Messor barbarus*. (*A*) Experimental paradigm. Four nestmates were kept together in a box. After four days, one ant was isolated and exposed to ambient air or increased levels of ozone for 2 h, and then returned to its nestmates. The behavior of the nestmates toward the reintroduced ant was recorded for 10 min. (*B*) Example traces of friendly or aggressive contacts toward reintroduced ants that were exposed to ambient air or different levels of ozone. Red, threat contact; gray, friendly contact; black, short contact (less than 1 s) or no contact. (*C*) Quantitative analysis of nestmates’ behaviors toward reintroduced ants. Aggression index = total time of threat contacts/total recording time. The experiment was performed with six colonies. Eight replicates per colony were tested with the reintroduced ant being either a nestmate exposed to ambient air or different ozone concentrations, or being a foreign conspecific ant. Box plots, median values and quartiles; whiskers, minimum and maximum values; dots, individual data points. Linear mixed model followed by post hoc pairwise comparisons between all treatments with Holm adjustment for multiple comparisons. Different letters indicate statistically different treatment groups. For all raw data of Fig. 1 and Dataset S1.

Cuticular hydrocarbons (CHCs) are essential for nestmate recognition in ants, serving to prevent robbery and parasitism from external sources, particularly by conspecifics from other colonies (26). Our previous studies demonstrated that ozone exposure could oxidize CHCs in fruit flies (23). This prompted us to investigate whether ozone exposure could alter nestmate recognition in ants. To address this, we first examined the harvester ant *Messor barbarus*, a cosmopolitan species that is easy to rear. After exposing a single worker to ozone, we reintroduced it to a box with nestmates and recorded the nestmates’ behavior (Fig. 1*A*). As a control, another worker was exposed to ambient air and afterward was similarly reintroduced to a box containing nestmates. Our results revealed that workers exposed to 100 ppb or higher levels of ozone elicited significantly more aggressive behaviors from their nestmates, including head-butting, biting, and threat displays, compared to those that were exposed to clean air instead (Fig. 1*B*, *SI Appendix*, Fig. S1*A*, and Movie S1). Interestingly, we hardly observed any aggression of the reintroduced ants toward their nestmates despite being threatened by them (Movie S2). To further evaluate the intensity of these aggressive interactions, we introduced a *M. barbarus* worker from a foreign colony (that revealed a similar but not identical CHC profile, *SI Appendix*, Fig. S3) into the arena. The introduction of foreign workers often elicited aggression that was much higher than that observed after the introduction of ozone-exposed nestmates (Fig. 1*A*). Unlike the latter, the foreign worker actively fought back (Movie S3). Our finding suggests that ozone exposure degrades some of the chemical cues essential for nestmate recognition, altering the dynamics of social interactions between nestmates of *M. barbarus*. To investigate whether the aggressive behavior elicited by ozone exposure is not specific to a single colony, we tested workers from five different *M. barbarus* colonies using the same behavioral protocol. Exposure to 100 ppb or higher levels of ozone consistently triggered aggressive behaviors from nestmates across all colonies tested (*SI Appendix*, Fig. S1 *B*–*F*). Interestingly, a combined analysis of all nests revealed already significant effects at only slightly increased levels of ozone of 50 ppb (Fig. 1*C*). Apparently, ozone exposure negatively affects nestmate recognition in *M. barbarus* ants, and this effect is not colony specific.

## Quantity of Alkenes in *M. barbarus* Decreases after Ozone Exposure.

**Fig. 2.**
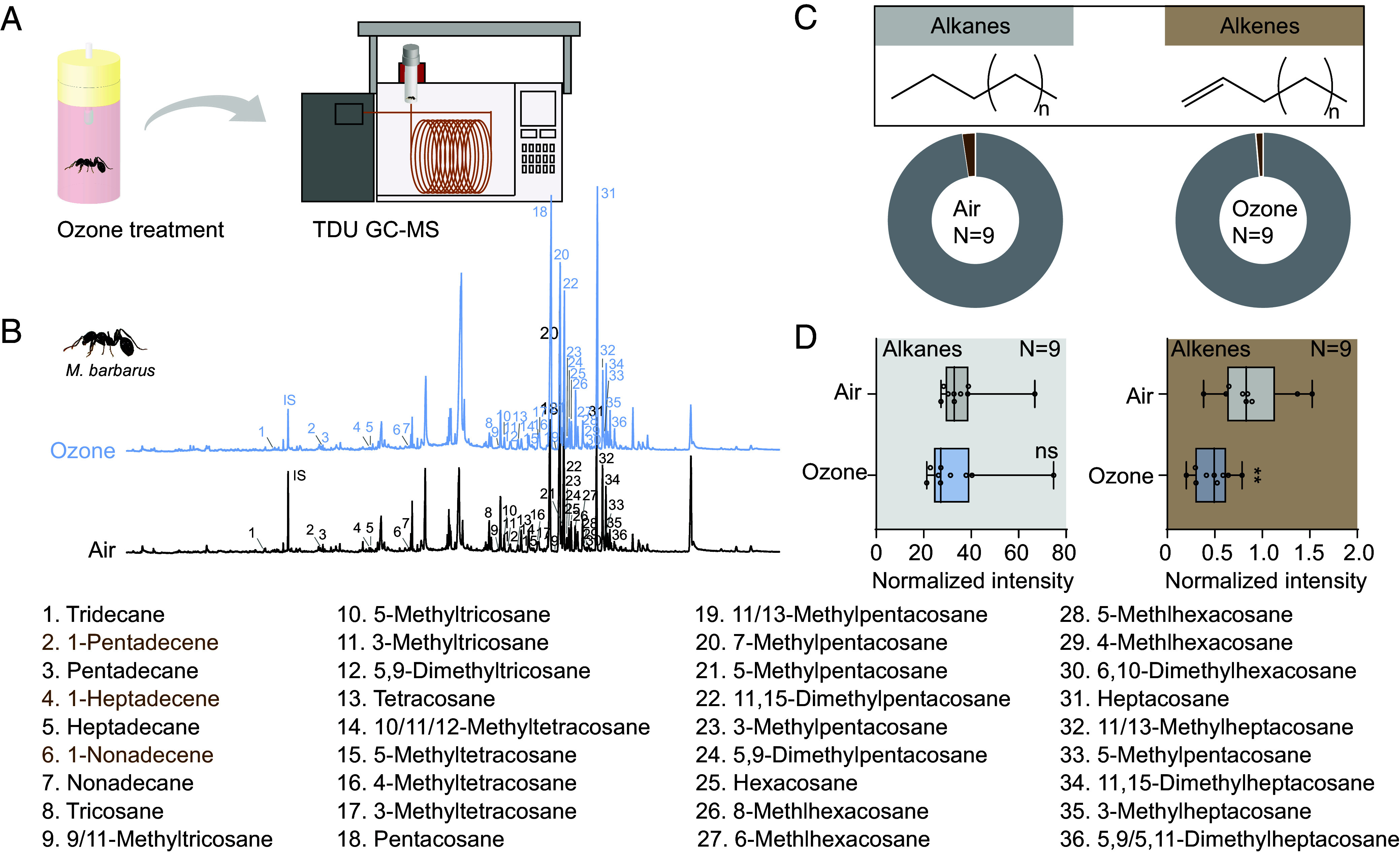
Cuticular hydrocarbon profile of *Messor barbarus*. (*A*) Experimental paradigm. An ant was either exposed to air or 100 ppb of ozone for 2 h before its CHC profile was analyzed by TDU GC-MS. (*B*) Example traces of an original (black) and an ozonated (blue) cuticular hydrocarbon profile of a *M. barbarus* worker with identified CHC compounds. Black names, alkanes; brown names, alkenes. IS indicates the internal standard. (*C*) Donut plot representation of total amount (i.e., peak area in GC) of alkanes vs. alkenes in two example ants that were exposed to ambient air (*Left*) or ozone (*Right*). For all identified compounds, see Dataset S2. (*D*) Quantitative analysis of alkanes (including methylated alkanes) and alkenes of workers exposed to ambient air (*Left*) or ozone (*Right*). Nine replicates were tested. Box plots, median values and quartiles; whiskers, minimum and maximum values; dots, individual data points. Two-side unpaired *t* test; ns, no significant difference; **P < 0.01. For all raw data of Fig. 2 and Dataset S1.

Ants possess a complex profile of cuticular hydrocarbons (CHCs) composed of multiple compounds that play an important role in chemical communication e.g., nestmate recognition. By using a gas chromatograph mass spectrometer coupled to a thermal desorption unit (TDU-GC-MS) we analyzed chemical profiles of *M. barbarus* workers that previously were either exposed to ambient air or to 100 ppb of ozone. We identified a total of 36 CHCs based on their mass spectra and those previously reported for *M. barbarous* (27, 28) (Fig. 2*A*). For further analysis, the 36 CHCs were broadly categorized into two groups—alkanes and alkenes (both including methylated branched alkanes or alkenes)—depending on whether their chemical structure included carbon–carbon double bonds or not (Fig. 2*B*). Overall, the quantity of alkenes in the CHC profile turned out to be low already when air-exposed ants were analyzed (Fig. 2*C*). However, it was further reduced when workers were exposed to ozone previously (Fig. 2*D*). Because the composition of alkanes that are known to be less-responsive with oxidants did not change upon ozone exposure (Fig. 2*D*), the above-described compromised nestmate recognition could be due to the ozone-induced degradation of alkenes (although we cannot exclude any potential additional effects of ozone on the behavior of ants). This is in line with the reported crucial role of alkenes for nestmate recognition in the ant *Formica exsecta* (29) that Z9-alkenes act as an important cues for nestmate recognition. Besides, alkenes also play a crucial role in honey bees (30). The finding that the level of aggression toward ozone-exposed ants did not reach that expressed toward foreign ants (Fig. 1*C*) may be due to the large quantity of alkanes and methylated alkanes unaffected by the ozone treatment (Fig. 2*D*).

## Ozone-Induced Aggression Is Conserved in Many Ant Species.

**Fig. 3.**
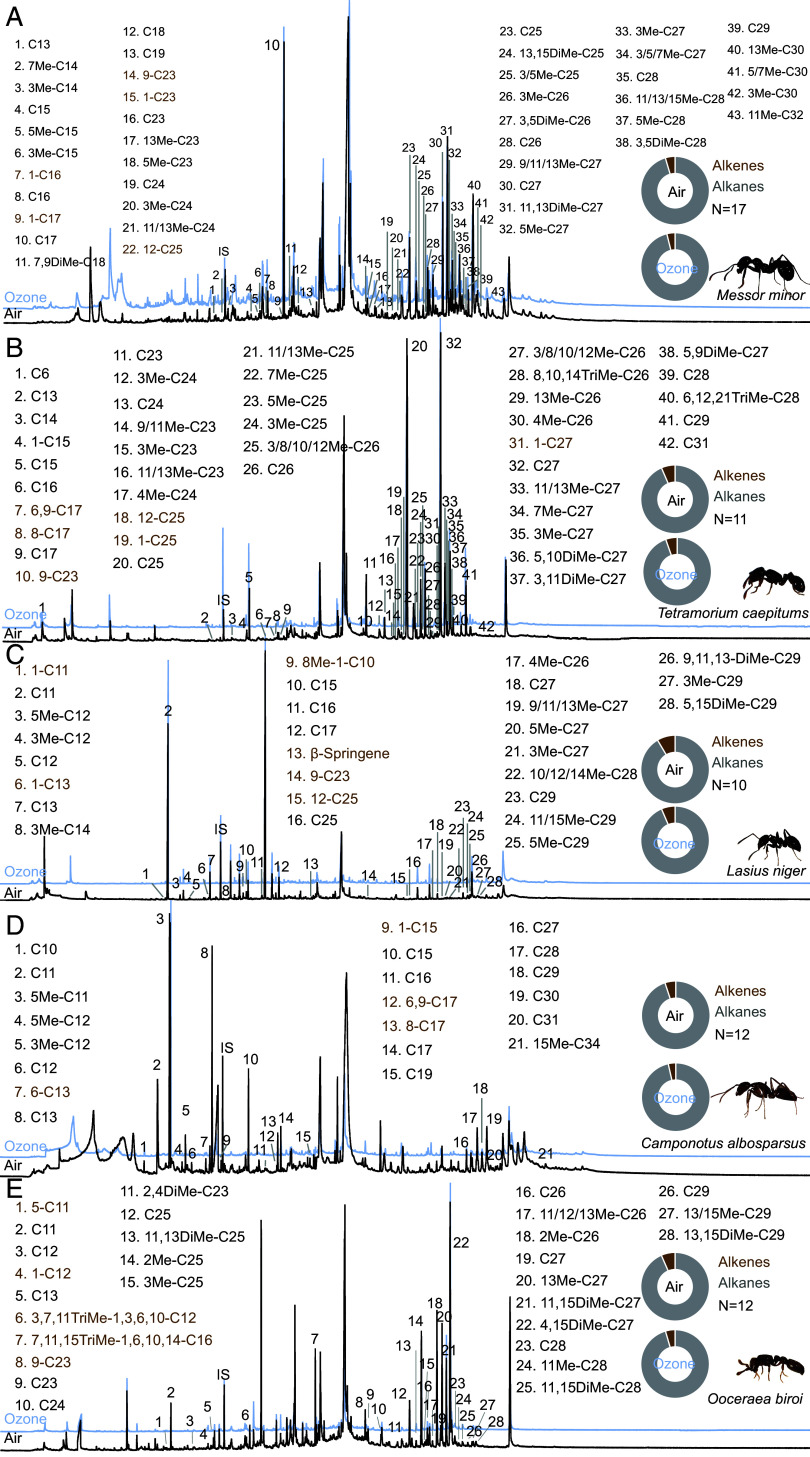
Example GC traces of a cuticular hydrocarbon profile of *Messor minor* (*A*), *Tetramorium caespitum* (*B*), *Lasius niger* (*C*), *Camponotus albosparsus* (*D*), and *Ooceraea biroi* (*E*) workers with identified CHC compounds. The black GC trace represents the CHCs of ants exposed to air, while the blue GC trace represents those exposed to ozone. Black names (abbreviated), alkanes; brown names (abbreviated), alkenes. IS indicates the internal standard. Donut plot representation of total amount (i.e., peak area in GC) of alkanes vs. alkenes in two example ants that were exposed to ambient air (*Top*) or ozone (*Bottom*). For all identified compounds, see Dataset S2.

To determine whether ozone exposure broadly affects social communication in ants, we examined both the behavior and chemical profiles of five species from the subfamilies Formicinae, Myrmicinae, and Dorylinae: *Messor minor*, *Camponotus albosparsus*, *Lasius niger*, *Tetramorium caespitum*, and *Ooceraea biroi*. We found most of the compounds that were previously found in profiles of those species [Fig. 3, (31–36); *SI Appendix*, Table S1].

**Fig. 4.**
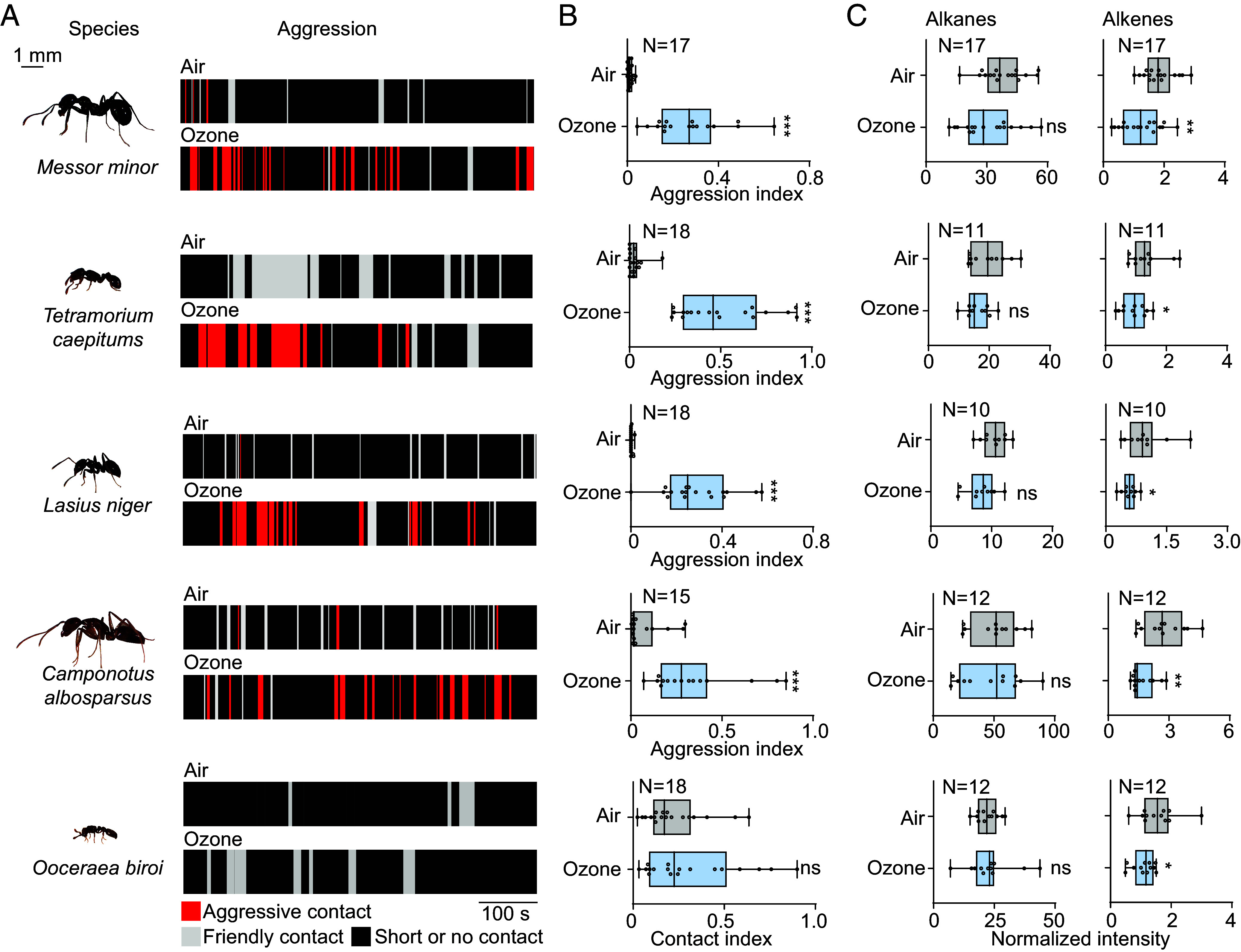
Ozone oxidizes alkenes in ants and often corrupts nestmate recognition. (*A*) Tested ant species and example traces of friendly or aggressive contacts toward reintroduced ants that were exposed to air of 100 ppb of ozone. For experimental paradigm, see Fig. 1*A*. Red, threat contact; gray, friendly contact; black, short contact (less than 1 s) or no contact. (*B*) Quantitative analysis of nestmates’ behaviors toward reintroduced ants. Aggression index = total time of threat contacts/total recording time. Seventeen replicates for *M. minor*; 18 replicates for *T. caespitum* and *L.niger*; 15 replicates for *C. albosparsus*. Contact index (for *O. biroi*), total contact time/total recording time. Eighteen replicates were tested. (*C*) Quantitative analysis of the ant’s alkanes and alkenes after being exposed to ambient air or 100 ppb of ozone. Box plots, median values and quartiles; whiskers, minimum and maximum values; dots, individual data points. Seventeen replicates for *M. minor*; 11 replicates for *T. caespitum*; 10 replicates for *L. niger*; 12 replicates for *C. albosparsus* and *O.biroi*. Two-side unpaired *t* test; ns, no significant difference; *P < 0.05; **P < 0.01; ***P < 0.001. For all raw data of Fig. 4 and Dataset S1.

Exposure to 100 ppb of ozone consistently reduced the quantity of alkenes across all tested species, while alkane levels remained unchanged (Fig. 4*C*). Correspondingly, ozone exposure induced aggression between nestmates in five out of six species (Fig. 4 *A* and *B* and Movies S4–S13). This suggests that alkenes play an important role in nestmate recognition in most ant species, despite their generally low abundance in CHC profiles. The finding that exposure to ozone in one species, *O. biroi*, induced changes of the CHC profile but did not result in any aggression between nestmates (Fig. 4 *A* and *B*) may be attributed to the unique biology of this species. In *O. biroi*, all individuals reproduce clonally via thelytokous parthenogenesis, and the colonies are queenless with no traditional division of labor regarding reproduction. At the same time, aggression levels between *O. biroi* ants belonging to different colonies have been observed to be low (37–39). Because ozone did not induce any aggression between nestmates in *O. biroi*, this species offered the possibility to look at other potential long-term detrimental effects at a colony level.

## Ozone Compromises Communication between Larvae and Nurses in *O. biroi*.

**Fig. 5.**
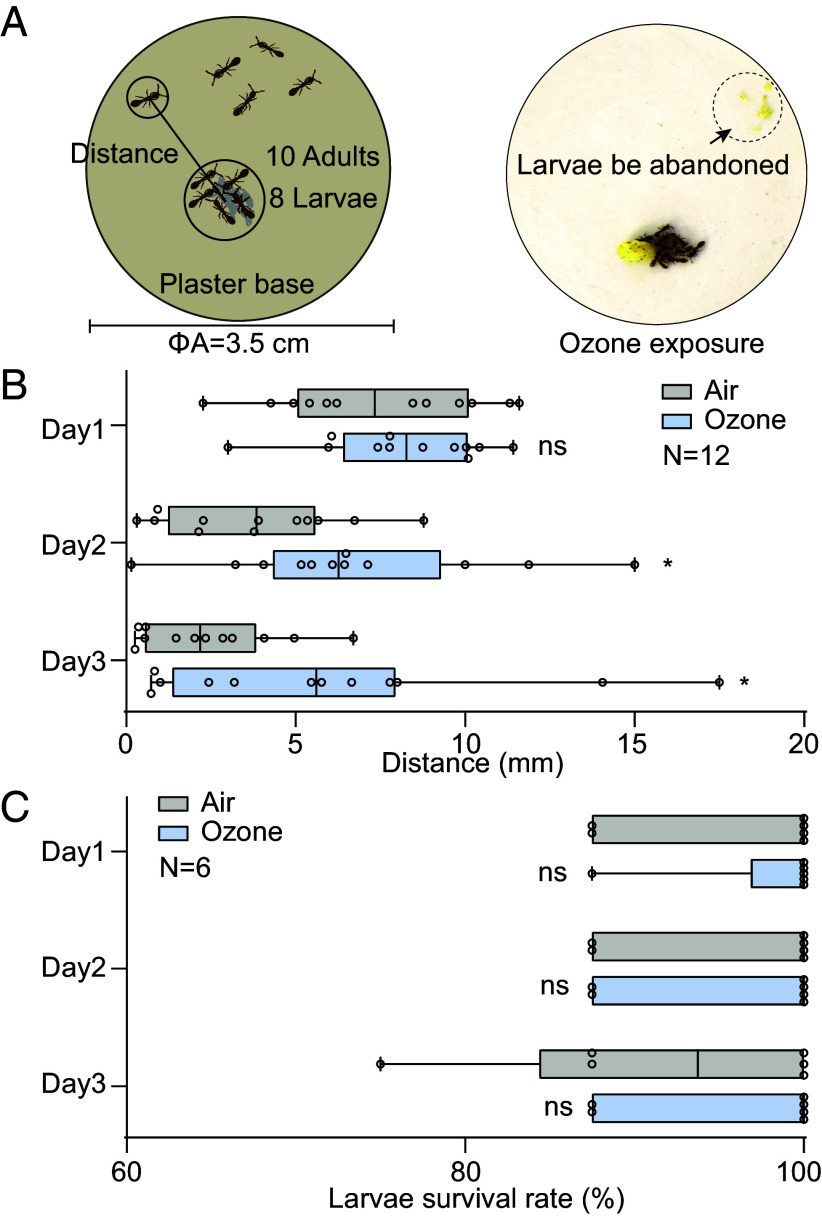
Ozone affects *O. biroi* social behavior at the colony level. (*A*) Experimental paradigm. (*Left*) 10 adult workers and eight larvae were put on a plaster base in a Petri dish with the workers immediately arranging the larvae in a narrow patch. Each 12 nests were either exposed to a flow of ambient air or to a flow of 100 ppb of ozone. Distance between each worker (including nurses staying with the larvae and foragers) and larval patch was measured every hour from 9 am to 6 pm. For each nest, distances of all measurements per day were averaged. (*Right*) Example of a larval patch completely abandoned by workers after ozone exposure. (*B*) Average distance of workers from larval patch per nest. 12 nests were tested. (*C*) Larval survival rate from day 1 to day 3 in the absence of workers. Six nests were tested. (*B* and *C*) Box plots, median values and quartiles; whiskers, minimum and maximum values; dots, individual data points. Two-sided unpaired *t* test, ns, not significant, *P < 0.05. For all raw data of Fig. 5 and Dataset S1.

We exposed small but functional colonies of *O. biroi* (i.e., 10 worker ants together with 8 third-instar larvae) over a period of 12 d to either ambient air or 100 ppb of ozone (Fig. 5*A*) and from 9 am to 6 pm recorded each ant’s position on an hourly base (Fig. 5*A*). Surprisingly, after 1 d of ozone exposure, we observed that adults discarded the majority of larvae in 3 out of 12 colonies (Fig. 5*B*). When measuring the distance between ants and larvae, we found that adults maintained significantly greater distances in the ozone-exposed colonies as compared to the colonies that were exposed to ambient air (Fig. 5 *B* and *C*). We investigated whether the workers’ neglect of the larvae in the presence of ozone was caused by larval death due to ozone poisoning. We therefore analyzed survival rates of air-exposed and ozone-exposed larvae in the absence of adult workers. To do so, eight larvae were placed on plaster and were exposed to either ambient air or 100 ppb of ozone. Our results showed that even after 4 d more than 70% of the larvae survived in both conditions, with no significant difference between air–and ozone exposure (Fig. 5*C*). This indicates that ozone has limited toxicity to larvae and suggests that larval abandonment (Fig. 5*B*) may be due to reduced communication between larvae and adults, although we cannot exclude that additional effects of ozone (e.g., a generally increased stress level, altered behavior, or a somehow affected immune system) might have been involved also. In summary, our data suggest that ozone exposure disrupts larval care behavior in adult *O. biroi*, potentially leading to developmental failure at the colony-level.

Several of the species tested in this study (*M. barbarus*, *M. minor*, *L. niger*, and *T. caespites* live close to human cities and forage during day time. One can therefore easily assume that foragers nowadays indeed can become exposed to the elevated levels of ozone that we used in our experiments. Contrary to that, workers of *Camponotus albosparsus* forage at night, when even close to cities ozone levels usually drop. This species therefore might not face such problematic ozone levels in its habitat. However, while ozone levels are known to drop after sun set, other oxidant pollutants like nitric oxides are more stable and might cause similar effects. Ozone levels are known to drop very close to ground surface (40) which potentially reduces the impact of ozone on ants foraging on the ground. However, many ant species (including some of our test species) are known to climb bushes or even trees during foraging. One can therefore expect that workers of these species face problematic levels of ozone. When it comes to *Ooceraea biroi*, this ant has become a widely used lab model and was therefore included in the analysis. With occupying the leaf litter of forests, where due to the high humidity, ozone levels can be expected to be rather low, the nests of this ant probably do not become affected by oxidant pollutants in their habitat. However, other ants like e.g. army ants (which were not available for our experiments) build their bivouac nests above ground and might, hence, easily face elevated levels of ozone within their colonies. As communication of the different ant species usually is based on chemical cues, we believe that, also we were not able to involve more species in our study, our findings might be valid for many ant species.

Eusocial insects, including ants, rely heavily on hydrocarbons on their body surface for nestmate recognition and social behavior. However, this chemically mediated sociality is increasingly threatened by oxidizing pollutants in modern environments. Here, we present an experimental framework to demonstrate how the air pollutant ozone affects social behavior in ants. Our findings show that elevated ozone levels oxidize alkenes in the cuticular hydrocarbons (CHCs) of multiple ant species and compromise nestmate recognition in five out of six tested species. Additionally, in the clonal raider ant *O. biroi*, ozone exposure compromised the care of adults for their larvae, highlighting its potential impact on insect societies at the colony level. Eusocial insects like ants, bees, and wasps fulfill important roles in their environment including ecosystem services like pollination in natural habitats (41) and agriculture (42), and control of pest insects (43). The functioning of these societies depends on nestmate recognition and social communication based on cuticular hydrocarbons. Although field studies already showed that oxidizing pollutants can disrupt the ecological service function of insects (44, 45), our findings that ozone can degrade some of the important chemical signals needed for nestmate recognition suggest that anthropogenic levels of oxidizing pollutants potentially may have far more negative consequences than previously feared.

## Materials and Methods

### Insect Collection and Husbandry.

Ant species used in this study included *Messor barbarus*, *Camponotus albosparsus*, *Lasius niger*, and *Tetramorium caespitum*, which were purchased from Antstore (https://www.antstore.net, Germany). Additionally, *Messor minor* and *Ooceraea biroi* (clonal lineage D) were obtained from Yuko Ulrich’s lab. The *O. biroi* colonies were reared in a plaster-based box and fed brood from *Messor* species under controlled conditions of 28 °C, 70% humidity, and a 12L:12D light/dark cycle. Other ant species were housed in tubes with a water reservoir blocked by cotton and fed with a diet consisting of the Bhatkar diet (46) and freeze-killed fruit flies (*Drosophila melanogaster* Canton-S). Two *Messor* species were fed on the Bhatkar diet and Vitakraft Menue-a mix of various plant seeds and dried mealworms. (Vitakraft Pet Care GmbH & Co. KG, Germany). Colonies were kept in fluon-coated boxes at 25 °C, 60% humidity, and a 12L:12D. All colonies were maintained over 8 mo, and each contained over 80 workers before ants were sampled for experiments.

### Ozone Exposure System.

The ozone exposure system was previously described (23, 24) (*SI Appendix*, Fig. S2). Briefly, ambient air served as the control air after being humidified to 70 ± 10% relative humidity. Ozone-enriched air was generated from the control air by passing it through an ozone generator (Aqua Medic, Germany). Different levels of ozonated air were prepared by mixing clean air with ozone-enriched air in a mixing box (a 100 L Plexiglas container). The ozonated experimental air was dynamically stored in the mixing box, from which the air was continuously monitored using an ozone monitor (BMT 932, BMT Messtechnik GmbH, Germany). Simultaneously, a flow of 0.2 L/min was directed into each of four 70 mL plastic vials containing individual ants (Experiments of figures 1–3) or to a box that contained 12 Petri dishes with *O. biroi* colonies (Experiment of figure 4). As a control, another set of four 70 mL vials or a box with 12 Petri dishes with *O. biroi* colonies was connected to the airflow of the control air.

### Behavioral Test for Nestmate Aggression.

Four ants were picked up randomly from a *M. barbarus* colony and then put into a fluon-coated plastic box (length, 8 cm, width, 5 cm, height, 10 cm) for 4 d with ad libitum supply of sugar water. After that, one ant (treatment ant) was randomly taken from that box, was exposed either to ambient air or to either 50 ppb, 100 ppb, 150 ppb, or 200 ppb of ozone for 2 h, and was then returned to its original box within 1 min. For each colony, 8 or 9 replicates were tested for each treatment. After recording, ants were not retested. We then recorded her nestmates’ behaviors for 10 min using a GoPro Camera 4, a Logitech C615 or a Xiaomi 12 T camera. Similar experiments were performed with ants of *M. minor*, *C. albosparsus*, *L. niger*, *T. caespitum*, and *O. biroi* colonies. Due to the smaller sizes of the latter two species, experiments with *T. caespitum* were performed with eight instead of four ants and experiments with *O. biroi* were performed with four ants but in a smaller arena (Petri dish, diameter 3.5 cm, height, 1 cm).

Each video was analyzed manually regarding the contacts of nestmates toward the reintroduced ant. Contacts like biting, head butting, or threatening with open mandibles were regarded as aggressive, while antennating with closed mandibles (which is regularly observed between nestmates) was regarded as friendly. We calculated an aggression index = total time of aggressive contacts/total recording time. As we never observed any aggression in *O. biroi*, we calculated for this species only a contact index (= total time of contact/total recording time). For all videos, we analyzed the first 10 min after the treatment ant was reintroduced to her nestmates. For *M. barbarus* we in addition quantified under the same conditions the aggression toward a conspecific ant of another colony. All behavioral experiments were performed at 25 °C and 70% humidity. Videos were analyzed by a person who was not informed about the treatment of the reintroduced ant.

### TDU GC-MS.

To avoid overloading the chemical profiles of ants during the TDU-GC-MS experiments, the smallest body-sized ant of each test species were selected and exposed to ozone (100 ppb) or ambient air for 2 h. For *Ooceraea biroi* (which all showed similar body sizes), we tested workers that were 13 d old after emerging from pupae. Immediately afterward, the ants were frozen at −20 °C for 30 min. For TDU-GC-MS analysis, individual ants were placed in microvials within thermal desorption tubes (GERSTEL, Germany). As an internal standard (IS), 0.5 μL of C10-Br (pure compound diluted 10^−3^ in hexane) was added to each microvial. Normalized intensity = identified compound peak area/ internal standard peak area. The thermal desorption tubes were loaded into a GERSTEL thermal desorption unit (TDU) using a GERSTEL MPS 2 XL multipurpose sampler. Desorption was carried out at 250 °C for 8 min, and the desorbed compounds were trapped at −50 °C in the liner of a GERSTEL CIS 4 Cooled Injection System, which utilized liquid nitrogen for cooling. The trapped components were then transferred to the gas chromatography (GC) column by heating the programmable temperature vaporizer injector at a rate of 12 °C/s to a final temperature of 270 °C, which was maintained for 5 min. The analysis was performed using a GC-MS system (Agilent GC 7890A coupled with an MS 5975C inert XL MSD unit; Agilent Technologies, USA) equipped with an HP-5 column (Agilent Technologies, USA). The GC oven temperature program started at 50 °C, held for 3 min, then increased at 15 °C/min to 230 °C, followed by an increase of 20 °C/min to 280 °C, which was held for 20 min. Mass spectra were acquired in electron ionization (EI) mode at 70 eV, covering a mass-to-charge (m/z) range of 33 to 500. All CHCs from different ant species were identified based on their MS spectrum.

### The Effect of Ozone at the Colony Level.

To test the effect of ozone at the colony level, we built artificial colonies by putting ten 15-d-old *O. biroi* workers together with eight 7-d-old larvae into a Petri dish (diameter, 3.5 cm) that was covered by plaster (Meyco Feinster Alabaster Modelliergips, b-boo baby & lifestyle GmbH, Germany mixed with water with the ratio of 2:1). It is a particular feature of *O. biroi* that such small colonies are functional, which has been established in past work (39). The borders of the Petri dishes were covered by mineral oil to prevent ants from escaping. Before the experiments, the plaster was soaked with water and the Petri dishes were placed in a box whose bottom was covered by wet cotton to continuously provide high humidity. Each box contained 12 Petri dishes with colonies and was connected either to a continuous flow of ambient air, or a continuous flow of 100 ppb of ozone. Photos (camera, Nikon D5300 coupled with the Nikon DX AF-S NIKKOR 18 to 55 mm, 1:3.5-5.6G II) of each colony were taken at every 1 h from 9 am to 6 pm and the distance between each adult and the larval cluster (when put together, adults always place all larvae in a dense cluster) was measured by using the Adobe Illustrator scale tool. Ants in the nest were defined as those in physical contact with the larvae. The nurse’s distance was considered zero (in some cases where there were no foragers, all worker distances were recorded as zero). The forager’s distance was measured relative to the nest. If more than 50% of the larvae were abandoned, the distance was calculated based on the nurse cluster and foragers in relation to the abandoned larvae cluster. All distances measured within one colony over the day were averaged to later compare worker–larval relationships in ozone- and air treated colonies. *Messor barbarus* brood was provided as food and water was added into the Petri dishes every two days. All behavioral experiments were performed at 25 °C and 70% humidity and lasted for 3 d.

In a second set of experiments, eight 7-day-old *O. biroi* larvae were placed on a humidified plaster-covered Petri dish (see above) and were kept in the absence of any workers. Six replicates were exposed to either a constant flow of 100 ppb ozone or to a flow of ambient air. To assess larval survival, every day a tiny brush was gently used to touch the larvae resulting in curvature behavior in living but not dead larvae. The survival rate of the larvae was recorded daily for three days.

### Statistical Analyses.

Statistical analyses (see the corresponding legends of each figure) and preliminary figures were conducted using GraphPad Prism v. 8 (GraphPad Software, USA). Figures were then processed with Adobe Illustrator CS5. Aggression indices of the six colonies of *M. barbarous* (Fig. 1*C*) were analyzed using a linear mixed-effects model with the *lme4* package in RStudio v. 2025.05.1. The model included Treatment (a six-level factor) as a fixed effect and Colony as a random effect to account for nonindependence of individuals within colonies. The response variable was cubic-root transformed to improve model fit. Model assumptions and residual diagnostics were assessed using the *DHARMa* package. The global effect of Treatment was tested with likelihood-ratio tests (function *drop1*), followed by post hoc pairwise comparisons between all levels of Treatment using the *emmeans* package, with Holm adjustment for multiple comparisons.

## Supplementary Material

Appendix 01 (PDF)

Dataset S01 (XLSX)

Dataset S02 (XLSX)

Movie S1. ***M. barbarus* reaction towards air-exposed nestmate**. 4 *M. barbarus* workers were kept in isolation for 4 days. After 4 days, one worker was isolated for 20 min, while being exposed to ambient air before it was reintroduced to the colony (ant marked in green). Behavior of the remaining three workers was scored.

Movie S2. ***M. barbarus* reaction towards ozone-exposed nestmate**. 4 *M. barbarus* workers were kept in isolation for 4 days. After 4 days, one worker was isolated for 20 min, while being exposed to slightly increased levels of ozone before it was reintroduced to the colony (ant marked in red). Behavior of the remaining three workers was scored.

Movie S3. ***M. barbarus* reaction towards foreing conspecific worker**. 4 *M. barbarus* workers were kept in isolation for 4 days. After 4 days, one worker was removed and after 20 min replaced by a conspecific worker from a foreign colony. Behavior of the remaining three workers was scored.

Movie S4. ***M. minor* reaction towards air-exposed nestmate**. 4 *M. minor* workers were kept in isolation for 4 days. After 4 days, one worker was isolated for 20 min, while being exposed to ambient air before it was reintroduced to the colony (ant marked in green). Behavior of the remaining three workers was scored.

Movie S5. ***M. minor* reaction towards ozone-exposed nestmate**. 4 *M. minor* workers were kept in isolation for 4 days. After 4 days, one worker was isolated for 20 min, while being exposed to slightly increased levels of ozone before it was reintroduced to the colony (ant marked in red). Behavior of the remaining three workers was scored.

Movie S6. ***T. caespitum* reaction towards air-exposed nestmate**. 4 *T. caespitum* workers were kept in isolation for 4 days. After 4 days, one worker was isolated for 20 min, while being exposed to ambient air before it was reintroduced to the colony (ant marked in green). Behavior of the remaining three workers was scored.

Movie S7. ***T. caespitum* reaction towards ozone-exposed nestmate**. 4 *T. caespitum* workers were kept in isolation for 4 days. After 4 days, one worker was isolated for 20 min, while being exposed to slightly increased levels of ozone before it was reintroduced to the colony (ant marked in red). Behavior of the remaining three workers was scored.

Movie S8. ***L. niger* reaction towards air-exposed nestmate**. 4 *L. niger* workers were kept in isolation for 4 days. After 4 days, one worker was isolated for 20 min, while being exposed to ambient air before it was reintroduced to the colony (ant marked in green). Behavior of the remaining three workers was scored.

Movie S9. ***L. niger* reaction towards ozone-exposed nestmate**. 4 *L. niger* workers were kept in isolation for 4 days. After 4 days, one worker was isolated for 20 min, while being exposed to slightly increased levels of ozone before it was reintroduced to the colony (ant marked in red). Behavior of the remaining three workers was scored.

Movie S10. ***C. albosparsus* reaction towards air-exposed nestmate**. 4 *C. albosparsus* workers were kept in isolation for 4 days. After 4 days, one worker was isolated for 20 min, while being exposed to ambient air before it was reintroduced to the colony (ant marked in green). Behavior of the remaining three workers was scored.

Movie S11. ***C. albosparsus* reaction towards ozone-exposed nestmate**. 4 *C. albosparsus* workers were kept in isolation for 4 days. After 4 days, one worker was isolated for 20 min, while being exposed to slightly increased levels of ozone before it was reintroduced to the colony (ant marked in red). Behavior of the remaining three workers was scored.

Movie S12. ***O. biroi* reaction towards air-exposed nestmate**. 4 *O. biroi* workers were kept in isolation for 4 days. After 4 days, one worker was isolated for 20 min, while being exposed to ambient air before it was reintroduced to the colony (ant marked in green). Behavior of the remaining three workers was scored.

Movie S13. ***O. biroi* reaction towards ozone-exposed nestmate**. 4 *O. biroi* workers were kept in isolation for 4 days. After 4 days, one worker was isolated for 20 min, while being exposed to slightly increased levels of ozone before it was reintroduced to the colony (ant marked in red). Behavior of the remaining three workers was scored.

## Data Availability

Study data are included in the article and/or supporting information.
