# Supplementary material for: Oxidizing pollutants can disrupt nestmate recognition in ants
Source: Proc Natl Acad Sci U S A. 2026 Feb 2;123(6):e2520139123. doi: 10.1073/pnas.2520139123 (PMC12890811; doi:10.1073/pnas.2520139123)
Supplement: Supplementary file 1 — Appendix 01 (PDF) [file pnas.2520139123.sapp.pdf]

Oxidising pollutants can disrupt nestmate recognition and division of labor in ants

Nan-Ji Jiang[1,2,5], Bhoomika Ashok Bhat[3], Eduardo Briceño Aguilar[1], Angela Lehmann[4], Yuko Ulrich[3], Bill S. Hansson[1,2,+], Markus Knaden[1,2,+,*]

Affiliations:

[1] Department of Evolutionary Neuroethology, Max-Planck Institute for Chemical Ecology

[2] Max Planck Center Next Generation Insect Chemical Ecology

[3] Social Behaviour Group, Max-Planck Institute for Chemical Ecology

[4] Workshop, Max-Planck Institute for Chemical Ecology

[5] Shenzhen Branch, Guangdong Laboratory of Lingnan Modern Agriculture, Key Laboratory of Synthetic Biology, Ministry of Agriculture and Rural Affairs, Agricultural Genomics Institute at Shenzhen, Chinese Academy of Agricultural Sciences, Shenzhen, China

[+] Authors share senior authorship

[*] Corresponding author: mknaden@ice.mpg.de

This supplementary file includes three figures (figure S1, S2, and S3), and movie legends (movie S1-S7).

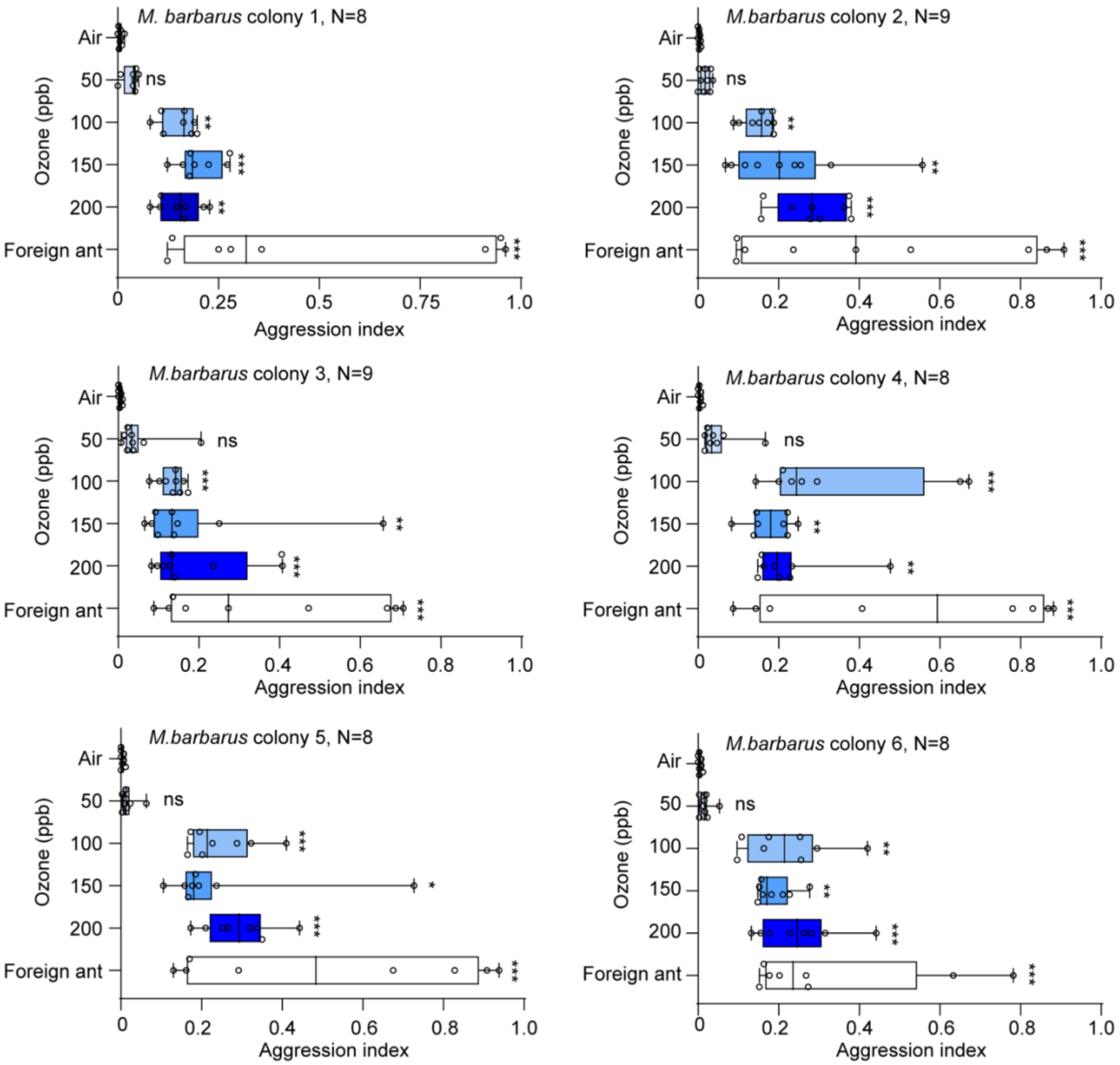

Fig S1. Quantitative analysis of nestmates' behaviors towards reintroduced ants in different *M. barbarus* colonies. a, Aggression index = total time of threat contacts/total recording time. Box plots, median values and quartiles; whiskers, minimum and maximum values; dots, individual data points. Kruskal Wallis test with Dunn's posthoc analysis for selected pairs (all groups tested against the air-exposed group); ns, no significant difference; *p<0.05; **p<0.01; ***p<0.001.

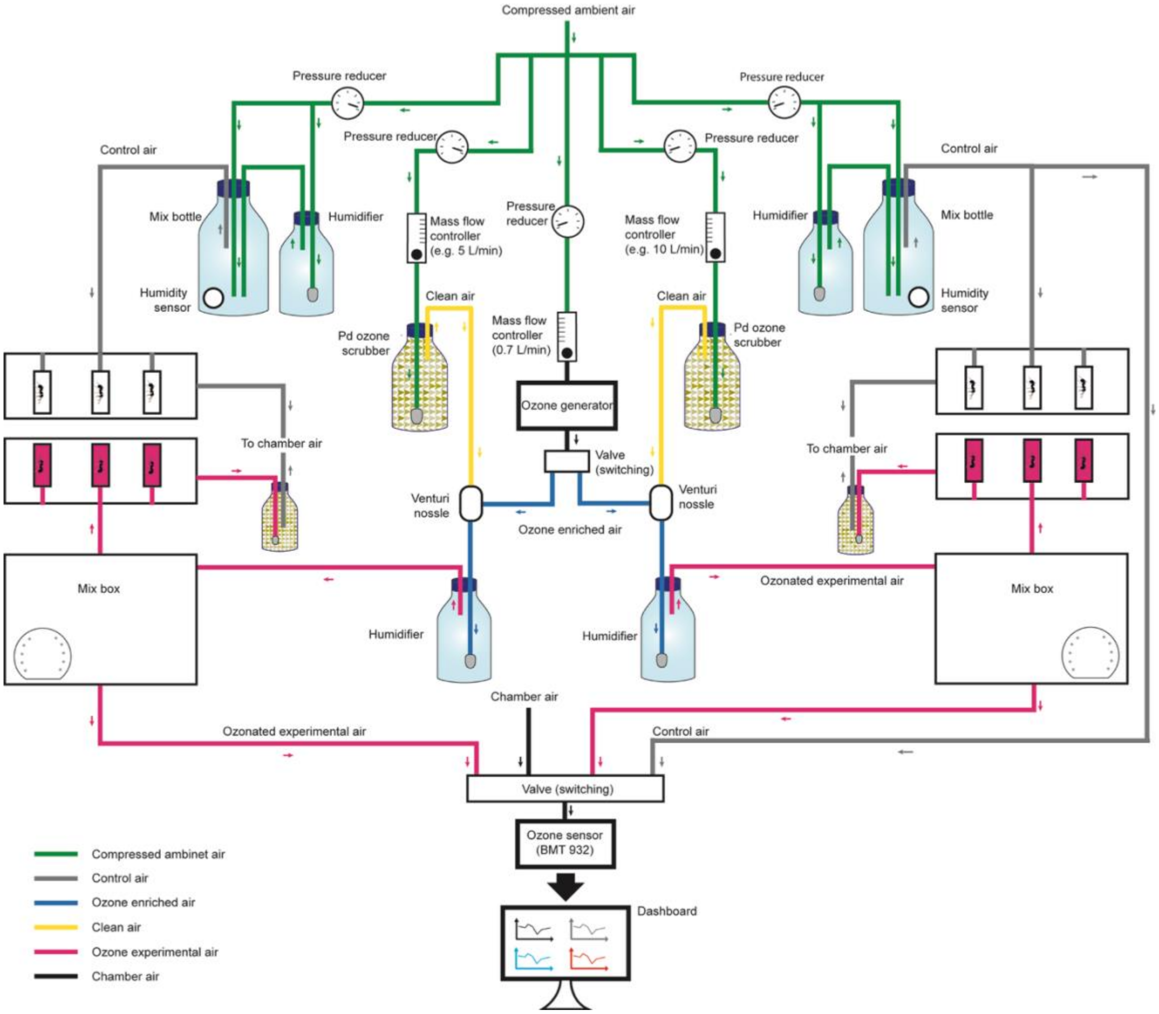

Fig S2. Schematic of the ozone device to produce defined levels of ozone. +

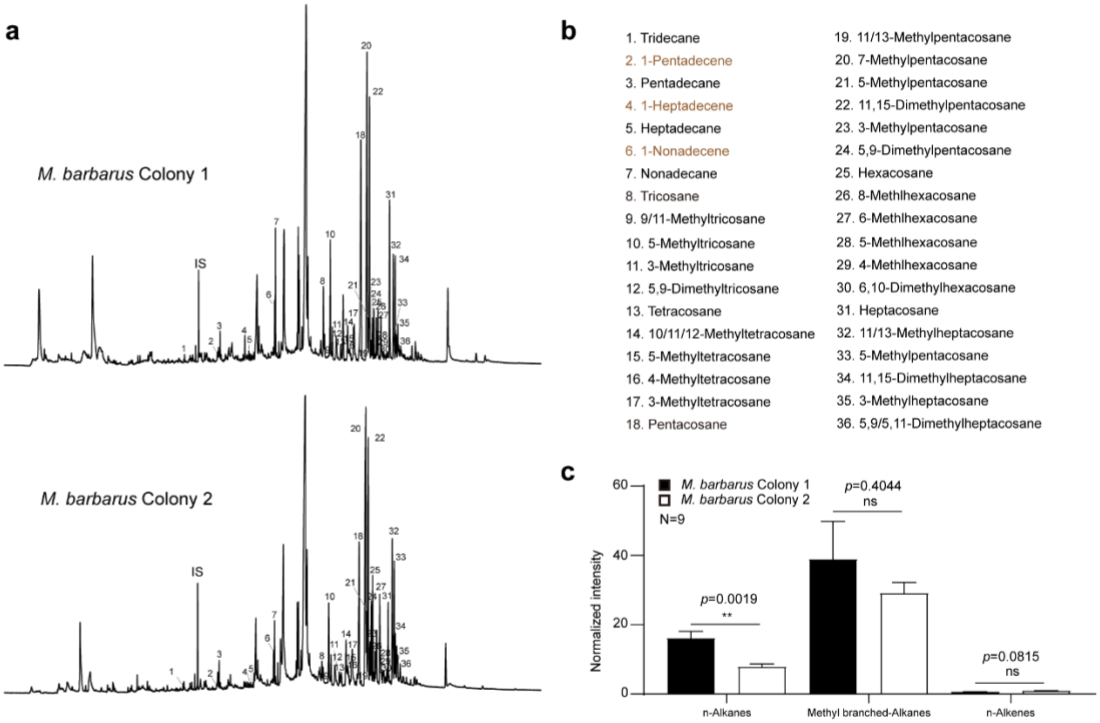

Figure S3. CHCs profiles from two different M. barbarus colonies. a, Ant CHC trace from colony 1 and colony 2. **b**, Identities of CHCs as suggested by MS analysis. **c,** Quantification of CHCs in three clusters n-alkanes, methyl branched alkanes, and alkenes.

Movie S1. *M. barbarus* reaction towards air-exposed nestmate. 4 *M. barbarus* workers were kept in isolation for 4 days. After 4 days, one worker was isolated for 20 min, while being exposed to ambient air before it was reintroduced to the colony (ant marked in green). Behavior of the remaining three workers was scored.

Movie S2. *M. barbarus* reaction towards ozone-exposed nestmate. 4 *M. barbarus* workers were kept in isolation for 4 days. After 4 days, one worker was isolated for 20 min, while being exposed to slightly increased levels of ozone before it was reintroduced to the colony (ant marked in red). Behavior of the remaining three workers was scored.

Movie S3. *M. barbarus* reaction towards foreing conspecific worker. 4 *M. barbarus* workers were kept in isolation for 4 days. After 4 days, one worker was removed and after 20 min replaced by a conspecific worker from a foreign colony. Behavior of the remaining three workers was scored.

Movie S4. *M. minor* reaction towards air-exposed nestmate. 4 *M. minor* workers were kept in isolation for 4 days. After 4 days, one worker was isolated for 20 min, while being exposed to ambient air before it was reintroduced to the colony (ant marked in green). Behavior of the remaining three workers was scored.

Movie S5. *M. minor* reaction towards ozone-exposed nestmate. 4 *M. minor* workers were kept in isolation for 4 days. After 4 days, one worker was isolated for 20 min, while being exposed to slightly increased levels of ozone before it was reintroduced to the colony (ant marked in red). Behavior of the remaining three workers was scored.

Movie S6. *T. caespitum* reaction towards air-exposed nestmate. 4 *T. caespitum* workers were kept in isolation for 4 days. After 4 days, one worker was isolated for 20 min, while being exposed to ambient air

before it was reintroduced to the colony (ant marked in green). Behavior of the remaining three workers was scored.

Movie S7. *T. caespitum* reaction towards ozone-exposed nestmate. 4 ***T. caespitum*** workers were kept in isolation for 4 days. After 4 days, one worker was isolated for 20 min, while being exposed to slightly increased levels of ozone before it was reintroduced to the colony (ant marked in red). Behavior of the remaining three workers was scored.

Movie. S8 *L. niger* reaction towards air-exposed nestmate. 4 *L. niger* workers were kept in isolation for 4 days. After 4 days, one worker was isolated for 20 min, while being exposed to ambient air before it was reintroduced to the colony (ant marked in green). Behavior of the remaining three workers was scored.

Movie. S9 *L. niger* reaction towards ozone-exposed nestmate. 4 *L. niger* workers were kept in isolation for 4 days. After 4 days, one worker was isolated for 20 min, while being exposed to slightly increased levels of ozone before it was reintroduced to the colony (ant marked in red). Behavior of the remaining three workers was scored.

Movie. S10 *C. albosparsus* reaction towards air-exposed nestmate. 4 *C. albosparsus* workers were kept in isolation for 4 days. After 4 days, one worker was isolated for 20 min, while being exposed to ambient air before it was reintroduced to the colony (ant marked in green). Behavior of the remaining three workers was scored.

Movie. S11 *C. albosparsus* reaction towards ozone-exposed nestmate. 4 *C. albosparsus* workers were kept in isolation for 4 days. After 4 days, one worker was isolated for 20 min, while being exposed to slightly increased levels of ozone before it was reintroduced to the colony (ant marked in red). Behavior of the remaining three workers was scored.

Movie. S12 *O. biroi* reaction towards air-exposed nestmate. 4 *O. biroi* workers were kept in isolation for 4 days. After 4 days, one worker was isolated for 20 min, while being exposed to ambient air before it was reintroduced to the colony (ant marked in green). Behavior of the remaining three workers was scored.

Movie. S13 *O. biroi* reaction towards ozone-exposed nestmate. 4 *O. biroi* workers were kept in isolation for 4 days. After 4 days, one worker was isolated for 20 min, while being exposed to slightly increased levels of ozone before it was reintroduced to the colony (ant marked in red). Behavior of the remaining three workers was scored.
